# Dementia challenges in Africa: Journeying through forgetfulness

**DOI:** 10.1002/hsr2.1561

**Published:** 2023-09-11

**Authors:** Burhan Kantawala, Najwa Zahra, Taha Oseili, Ali Jawad, Souhail Ouardouz, Abubakar Nazir, Magda Wojtara, Olivier Uwishema

**Affiliations:** ^1^ Oli Health Magazine Organization, Research and Education Kigali Rwanda; ^2^ Faculty of General Medicine Yerevan State Medical University Yerevan Armenia; ^3^ Faculty of Medicine Beirut Arab University Beirut Lebanon; ^4^ Faculty of Medicine Damascus University Damascus Syria; ^5^ Faculty of Medicine and Pharmacy Cadi Ayyad University Marrakech Morocco; ^6^ Department of Medicine King Edward Medical University Lahore Pakistan; ^7^ Department of Medicine University of Michigan Medical School Ann Arbor Michigan USA; ^8^ Department of Medicine Clinton Global Initiative University New York New York USA; ^9^ Faculty of Medicine Karadeniz Technical University Trabzon Turkey

**Keywords:** Alzheimer's disease, dementia, lewy body dementia, aging, Parkinson disease

## 1 INTRODUCTION

Dementia encompasses a wide spectrum of conditions characterized by a gradual deterioration of cognitive abilities, including memory, language, and thinking skills, to the extent that it significantly impairs a person's ability to perform everyday activities. The global impact of dementia is substantial, affecting the overall health and well‐being of individuals, placing significant strain on healthcare systems and caregivers, influencing social dynamics, and driving research and innovation. Addressing the challenges posed by dementia requires collective efforts to improve care, support, and resource allocation. Consequently, it is imperative to study dementia in Africa due to the region's growing elderly population, limited healthcare resources, socioeconomic disparities, and varying cultural beliefs. By focusing on dementia in Africa, it becomes possible to effectively address the growing burden of this disease, tailor interventions to meet specific, local needs, and contribute to the advancement of global knowledge in dementia research and care.^1^

The article's primary objectives encompass accentuating dementia's global significance and its unique challenges within Africa, with a focus on targeted interventions. It seeks to spotlight factors contributing to dementia prevalence in the region, notably cardiovascular risks and cultural perceptions, while emphasizing the necessity for comprehensive strategies. The paper addresses the stigma attached to dementia, the scarcity of healthcare resources and infrastructure, and the pressing need for awareness and support. Additionally, it spotlights the escalating dementia incidence in specific African areas, urging further research, assistance initiatives, and preventive actions to counter its effects.

## 2 PREVALENCE AND BURDEN OF DEMENTIA IN AFRICA

As the aging population in low and middle‐income countries continues to rise, dementia poses an escalating challenge for the African continent. The prevalence of dementia ranges from 2.3% to 20.0%, and incidence rates are reported at 13.3 per 1000 person‐years, coupled with a rising mortality rate in rapidly developing parts of Africa.^1^ Multiple factors contribute to the high burden of dementia, including cardiovascular risk factors (e.g., hypertension, diabetes, dyslipidemia) as well as advancing age. Lifestyle choices such as smoking, unhealthy diet, lack of physical activity, and excessive alcohol consumption also play a role in increasing the burden of dementia.^1^

Dementia places a heavy burden on individuals, families, and communities, gradually diminishing cognitive function and daily abilities, causing frustration and dependency, causing emotional and financial challenges for caregivers, and causing healthcare costs and productivity loss to the communities to develop support systems for those with dementia.

## 3 CULTURAL AND SOCIAL PERSPECTIVES ON DEMENTIA IN AFRICA

The limited research conducted in South Africa indicates that certain cultural groups perceive dementia as a form of divine punishment or curse resulting from witchcraft or being bewitched. Traditional healers, in these contexts, are believed to have the ability to cure dementia. These perceptions lead to suspicion and fear toward the behavioral and psychosocial symptoms associated with dementia. Although reports of negative responses and intense aggression against individuals with dementia who are labeled as witches or bewitched are currently anecdotal, media reports suggest that such incidents occur. The negative attitudes and discriminatory behavior toward individuals with dementia and their families have far‐reaching implications globally. These implications include challenges in social exclusion and feelings of isolation. The personalized stigma associated with dementia can lead to a sense of shame, concealment, and limited disclosure. Individuals may also feel compelled to hide their vulnerabilities, while families may experience resentment, guilt by association, and self‐imposed isolation. The social avoidance, discrimination, and loneliness that result from these attitudes can lead to dehumanization when people stop visiting. Furthermore, there may be anxiety about harmful belief systems.^2^ It is imperative to promote awareness across different age groups to motivate younger generations to take action toward finding a disease‐modifying therapy, enhancing care, and elevating the standard of living for individuals with dementia. By raising awareness, younger generations can be empowered to engage proactively in advocating for the needs of individuals with dementia and their families. This, in turn, can lead to the development of innovative solutions and interventions that can improve the lives of those affected by dementia.^3^

## 4 CHALLENGES IN DEMENTIA CARE IN AFRICA

**Figure 1:**
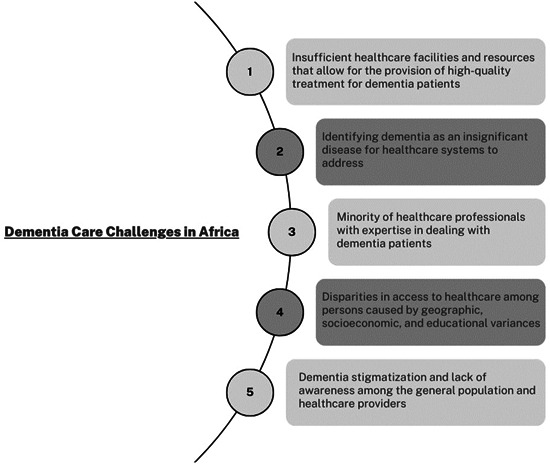
Challenges associated with dementia care in Africa.

With the so‐called “Epidemiological Transition” that Africa is going through, and with the increase in the age of living people such transition has influenced an increase in the prevalence of dementia in Africa as stated by the latter in the article. However, these developments are being hampered by a lack of infrastructure and healthcare resources to meet the needs of persons with dementia. Africa has a scarcity of dementia specialists to this day, and those that do exist are mostly concentrated in urban regions. In a cross‐sectional study conducted in rural Uganda, healthcare personnel who were not trained in mental health were able to identify cases of dementia but were unable to advance due to a lack of adequate training.^4^ Furthermore, several healthcare practitioners have identified dementia in patients as a natural aging condition. This only proves that dementia is still a misconstrued subject in Africa.^4^ Finally, research undertaken by WHO in partnership with the INDEPTH network of Health and Demographic Surveillance Sites has highlighted health disparities in dementia, such as gender, socioeconomic level, and place of residence.^5^ In Ghana, city dwellers and those with a higher socioeconomic status were among the most important predictors of healthcare utilization when compared to rural residents. In rural South Africa, dementia patients and those with lower socioeconomic status died at a higher rate than in urban regions. This only brings us to a conclusion on the various challenges that Africa currently faces regarding addressing dementia (Figure 1).

## 5 CONCLUSION: RECOMMENDATIONS, INITIATIVES, AND STRATEGIES FOR DEMENTIA CARE IN AFRICA

It is crucial to design efficient ways of life, dietary, and medication interventional and clinical methods to achieve achieving the greatest health impact, even though it is crucial to find new knowledge, especially from genetically distinct African people. Stroke and other cardiovascular diseases are on the rise in Africa, making it crucial to minimize risk factors globally. The biggest reversible risk factor of ischemia attacks among Africans is hypertension. Among the other factors that can be changed include diabetes, dyslipidemia, lack of activity, and poor eating habits. Additionally, conditions and comorbidities associated with health that are likely to raise the risk of changes in the individuals' mental and physical health should be monitored continuously.^1^ Such a comprehensive strategy to address rises in the incidence of dementia should take into account the impact on the expanding number of providers who will be needed to assist a rising percentage of persons with dementia. Evidence‐based interventions to help caregivers should therefore be included in any such strategy.^6^

**Figure 2:**
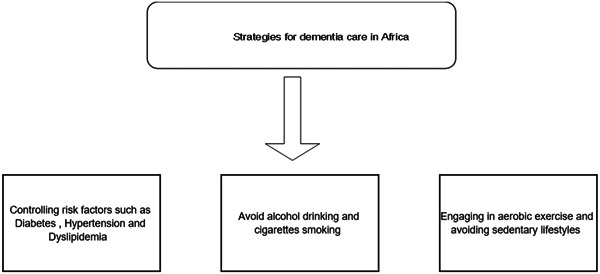
Strategies for dementia care in Africa.

An investigation into the projected incidence and prevalence of dementia has revealed an alarming increase in its occurrence attributable to several contributing factors. Findings indicate that the countries primarily vulnerable to this surge are predominantly situated in the northern regions of Africa.^6^, ^7^ To help with dementia patient care in the impoverished nations of Africa without advanced technology and appropriate medication, further studies and assistance programs are required. Despite the lack of accurate data, it is clear that decreasing risk factors and implementing prevention measures will help to lower mortality and morbidity rates (Figure 2).

## AUTHOR CONTRIBUTIONS

**Burhan Kantawala**: Writing—original draft; writing—review and editing. **Najwa Zahra**: Writing—original draft; writing—review and editing. **Taha Oseili**: Writing—original draft; writing—review and editing. **Ali Jawad**: Writing—original draft; writing—review and editing. **Souhail Ouardouz**: Writing—original draft; writing—review and editing. **Abubakar Nazir**: Supervision; validation; visualization; writing—original draft; writing—review and editing. **Magda Wojtara**: Writing—original draft; writing—review and editing. **Olivier Uwishema**: Writing—original draft; writing—review and editing.

## CONFLICT OF INTEREST STATEMENT

The authors declare no conflict of interest.

## TRANSPARENCY STATEMENT

The lead author Abubakar Nazir, Olivier Uwishema affirms that this manuscript is an honest, accurate, and transparent account of the study being reported; that no important aspects of the study have been omitted; and that any discrepancies from the study as planned (and, if relevant, registered) have been explained.

## Data Availability

The authors have nothing to report.
